# Alterations in zinc, copper, and iron levels in the retina and brain of Alzheimer's disease patients and the APP/PS1 mouse model

**DOI:** 10.1093/mtomcs/mfae053

**Published:** 2024-11-08

**Authors:** Seyed Mostafa Hosseinpour Mashkani, David P Bishop, Mika T Westerhausen, Paul A Adlard, S Mojtaba Golzan

**Affiliations:** Institute for Biomedical Materials and Devices, School of Mathematical and Physical Sciences, Faculty of Science, University of Technology Sydney, Sydney, NSW 2007, Australia; Hyphenated Mass Spectrometry Laboratory, School of Mathematical and Physical Sciences, University of Technology Sydney, Broadway, Sydney, NSW 2007, Australia; Hyphenated Mass Spectrometry Laboratory, School of Mathematical and Physical Sciences, University of Technology Sydney, Broadway, Sydney, NSW 2007, Australia; Synaptic Neurobiology Laboratory, The Florey Institute of Neuroscience and Mental Health, The University of Melbourne, Melbourne 3010, Australia; Vision Science Group (Orthoptics Discipline), Graduate School of Health, University of Technology Sydney, Sydney, NSW 2007, Australia

**Keywords:** Alzheimer's disease, aging, retina, transition metals, solution nebulization–inductively coupled plasma–mass spectrometry, laser ablation–inductively coupled plasma–mass spectrometry

## Abstract

Transition metals like copper (Cu), iron (Fe), and zinc (Zn) are vital for normal central nervous system function and are also linked to neurodegeneration, particularly in the onset and progression of Alzheimer's disease (AD). Their alterations in AD, identified prior to amyloid plaque aggregation, offer a unique target for staging pre-amyloid AD. However, analysing their levels in the brain is extremely challenging, necessitating the development of alternative approaches. Here, we utilized laser ablation–inductively coupled plasma–mass spectrometry and solution nebulization–inductively coupled plasma–mass spectrometry to quantitatively measure Cu, Fe, and Zn concentrations in the retina and hippocampus samples obtained from human donors (i.e. AD and healthy controls), and in the amyloid precursor protein/presenilin 1 (APP/PS1) mouse model of AD and wild-type (WT) controls, aged 9 and 18 months. Our findings revealed significantly elevated Cu, Fe, and Zn levels in the retina (*P < .05, *P* < .01, and *P* < .001) and hippocampus (*P < .05, *P < .05, and *P < .05) of human AD samples compared to healthy controls. Conversely, APP/PS1 mouse models exhibited notably lower metal levels in the same regions compared to WT mice—Cu, Fe, and Zn levels in the retina (**P < .01, *P < .05, and *P < .05) and hippocampus (**P < .01, **P < .01, and *P < .05). The contrasting metal profiles in human and mouse samples, yet similar patterns within each species’ retina and brain, suggest the retina mirrors cerebral metal dyshomoeostasis in AD. Our findings lay the groundwork for staging pre-AD pathophysiology through assessment of transition metal levels in the retina.

## Graphical Abstract

**Graphical Abstract:**
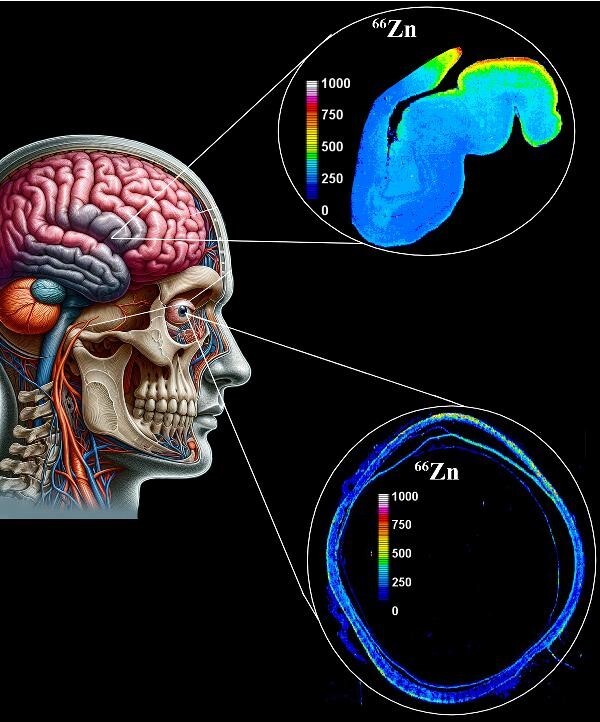

## Introduction

Alzheimer's disease (AD), a progressive neurodegenerative disorder marked by memory loss and behavioural changes, is often linked with aging [1]. Recent focus has shifted to the analysis of transition metals [copper (Cu), iron (Fe), and zinc (Zn)] in the brain, as their age-related distribution and concentration alterations could serve as a potential diagnostic marker for AD [2].

It has been reported that brain metal dyshomoeostasis is associated with normal aging, but it is further exacerbated in several neurodegenerative disorders, including Alzheimer's disease and Parkinson's disease. This exacerbation results in excessive reactive oxygen species generation and the formation of amyloid-beta (Aβ) plaques and neurofibrillary tangles (NFTs), which are pathological hallmarks of AD [3–5]. Clinical diagnoses and autopsy findings have revealed elevated concentrations of these metals in Aβ plaques in AD patients, supporting the ‘metals hypothesis of AD’, which posits that a balance of transition metals is crucial for neuronal function [6]. Despite advancements in research, a key question remains: Do age-related changes in metal ions differ between healthy individuals and those with AD, and if so, to what extent?

Currently, neuroimaging tools such as computed tomography [7], magnetic resonance imaging [8, 9], and positron emission tomography [9] are used for identifying biomarkers of AD pathology. However, these methods are expensive and often diagnose AD at advanced stages. Consequently, the retina, as an extension of the brain, is increasingly being explored as a non-invasive and accessible site for early AD detection [10]. Aβ plaques, a primary pathological feature of AD, have been identified in the retinas of patients, especially in early-stage cases [11–14].

Hyperspectral retinal imaging (HSRI) is a label-free, non-invasive technique for measuring Aβ. HSRI leverages the influence of Aβ on light scattered wavelengths between 460 and 570 nm, resulting in increased Rayleigh scattering due to the presence of Aβ [15, 16]. Furthermore, curcumin, a natural fluorophore that binds to Aβ, was used for labelling Aβ and measuring retinal fluorescence *in vivo* [17, 18]. Abnormal tau, characterized by hyperphosphorylated tau and its inclusion in NFTs as the second prominent indication of neuropathology in AD, has been reported in retinal layers, particularly the plexiform layer, inner nuclear layer, and ganglion cell layer of post-mortem retinas of confirmed AD cases [19–21].

Consistent with human findings, pathological features of AD have been reported in the retinas of various animal models. Soluble and insoluble forms of Aβ have been identified in the retina of sporadic models and transgenic mice harbouring familial AD mutations [12, 22–25]. Furthermore, amyloid precursor protein (APP), the precursor of Aβ protein, has been found in retinas of ADtg drosophila, various ADtg mice (Tg2576, hTgAPP^tg/tg^, APP_SWE_/PS1_ΔE9_, and APP_SWE_/PS1_M146L/L286V_), and the naturally occurring sporadic rodent strain *Octodon degus* [26–29]. Moreover, intracellular aggregates of pTau were identified in retinas of the APP_SWE_/PS1_M146L/L286V_ mouse model of AD [30].

In this study, we investigate the potential of monitoring age-related changes in cerebral Cu, Fe, and Zn concentrations through the eyes of APP/presenilin 1 (APP/PS1) and wild-type (WT) mice aged 9–18 months. Subsequently, we extend our analysis to post-mortem human donors, comparing Cu, Fe, and Zn levels in the eyes and brain tissues of both AD patients and post-mortem subjects without AD history. Our research aims to provide insights into the correlation between age-associated metal alterations in the retina and brain in AD, potentially bridging the gap in early AD diagnosis.

## Materials and methods

All experiments were conducted within the Graduate School of Health at the University of Technology Sydney. APP/PS1 and normal aged mice were obtained from the Florey Institute of Neuroscience and Mental Health. All animal experimental procedures were approved by the Florey Institute of Neuroscience Animal Ethics Committee prior to the commencement of experiments (19-060-FINMH). AD and healthy control human brain (i.e. hippocampus) and eye samples were obtained from the Netherlands Brain Bank. A biosafety approval was sought and obtained from UTS prior to commencement of experiments: “(2016-05-R-G) Implications of retinal neurodegeneration in Alzheimer’s Disease.”

### Human samples

A total of nine AD and six healthy control samples were obtained. The mean age of human AD and age-matched healthy donors was 75 ± 10 and 85 ± 10, respectively (Table 1).

**Table 1.** Demographics of human samples

	AD	Healthy control
Age	75 ± 10	85 ± 10
Gender (F/M)	6/3	1/5
Brain weight (g)	1120 ± 131	1260 ± 79
Post-mortem delay (h)	5.5 ± 3	7 ± 2
pH	6.5 ± 0.5	6.5 ± 0.5

### Animal samples

APP/PS1, a double transgenic mouse expressing a chimeric mouse/human APP (Mo/HuAPP695swe) and a mutant human presenilin 1 (PS1-dE9), and age-matched C57BL6 WT mice were used in our experiments. We chose to examine animals at 9 and 18 months of age, as this reflects Aβ deposition in the hippocampus, cognitive impairment, and also impaired long-term potentiation in the CA1 region of the hippocampus.

#### Tissue collection

A total of 20 mice with an equal gender distribution were included in this study (10 APP/PS1 and 10 WT—5 males/5 females in each group). After sacrificing animals using sodium pentobarbitone (80 mg/kg), transcardial perfusion was performed using 0.1 M phosphate buffer saline (PBS). The brain and whole eyes were removed and immediately placed in paraformaldehyde (4% w/v) and stored at 4°C overnight. They were then cryopreserved in a 30% sucrose solution (PBS) for 3 days. Tissues were finally placed in an appropriate size mould and filled with optimal cutting temperature compound and stored at −80 °C. Tissues were sectioned using the Leica CM1950 (Leica Biosystems) at a thickness of 10 μm. For solution nebulization–inductively coupled plasma–mass spectrometry (SN–ICP–MS), the hippocampus, cortex and the retina were dissected by following the protocols outlined in [31].

### Haematoxylin and eosin of human paraffin-embedded tissue

Paraffin slides were deparaffinized with xylene, rehydrated through graded alcohols, and washed with deionized water. Then, they were stained with haematoxylin for 3 min and eosin for 30 s before being re-immersed in alcohol and xylene. The slides were coverslipped and dried overnight in a fume hood [32].

### Laser ablation–inductively coupled plasma–mass spectrometry 

Laser ablation–inductively coupled plasma–mass spectrometry (LA–ICP–MS) was employed to measure the concentration of Cu, Fe, and Zn and their spatial distribution in the brain and retina of post-mortem subjects with AD history and post-mortem subjects without AD history samples. Three slices from each sample (brain and eye) were ablated and analysed by LA–ICP–MS. The study was carried out on an Elemental Scientific Lasers NWR193 laser hyphenated to an Agilent Technologies 7700 ICP–MS, with 3 ml/min H_2_ added in the reaction cell [33], and argon used as the carrier gas. LA–ICP–MS conditions were optimized on NIST 612 Trace Element in Glass CRM. The samples were ablated with a 50-µm spot size and a scan speed of 200 µm/s at a frequency of 20 Hz. Quantification was performed via the use of external matrix-matched standards [34].

### Solution nebulization–inductively coupled plasma–mass spectrometry

Solution nebulization–inductively coupled plasma–mass spectrometry (SN–ICP–MS) was used to analyse digested WT, and APP/PS1 mice tissue (retina, hippocampus, and cortex) and standards; 500 µl of HNO_3_ 69% (SEASTAR, Choice Analytical) was added to an equal weight of freeze-dried mouse model samples (retina, hippocampus, and cortex). The samples were allowed to digest overnight and then diluted to a final volume of 2 ml with Milli-Q water. SN–ICP–MS was performed using 7700x series ICP–MS (Agilent Technologies, Waldbronn, Germany), which was equipped with a Micromist^TM^ concentric nebulizer (Glass Expansion, West Melbourne, Australia) and a Scott-type double-pass spray chamber. All experiments used 99.9995% ultra-high purity liquid argon (Argon 5.0, Coregas Pty Ltd, Yennora, NSW, Australia). Solution-based samples were transferred to the ICP–MS using a 1.02-mm internal diameter Tygon tubing and a three-channel peristaltic pump. The solutions were pumped at a continuous flow rate of 1.0 ml/min. All digested samples were analysed against a 6-point calibration curve using multielemental liquid standards 100 μg/ml supplied by Choice Analytical (Thornleigh, New South Wales, Australia) on an Agilent 7700 ICP–MS (Agilent Technologies, Mulgrave, Australia) in single quadrupole mode equipped with a Micromist nebulizer and a Scott-type double-pass spray chamber cooled to 2°C for sample introduction. A 100 μg/l rhodium solution in 1% HNO_3_ was used as an internal standard and introduced into the analyte flow via a T-connector post-pump, and platinum sampling and skimmer cones were used.

### Image analysis

The data were collated into a single image file using in-house developed software, Pew^2^ [35], and imported into ImageJ (NIH, USA). A contour was drawn on the boundary of all regions of interest (hippocampus, cortex, and retina). The Allen Mouse Brain Atlas was used as a reference [36]. 4′,6-diamidino-2-phenylindole (DAPI) staining was also used to visualize the gross anatomical morphology and to better guide the process of identifying regions of interest. Following this process, the mean grey intensity value of each region was measured using the ImageJ built-in function. A minimum of three images per organ per region were analysed and the average value taken as representative mean metal load.

### Statistical analysis

All statistical analysis was performed using Graphpad Prism (Dotmatics, USA). Results are presented as mean ± standard error of the mean (SEM). Normality of data distribution was assessed using the D'Agostino & Pearson test. An unpaired *t-*test was used to compare differences between the two animal groups for each of the metals and anatomical regions.

## Results

### Evaluation of Fe, Cu, and Zn in the eye and brain (WT and APP/PS1 mice models)

#### Copper

**Figure 1.**
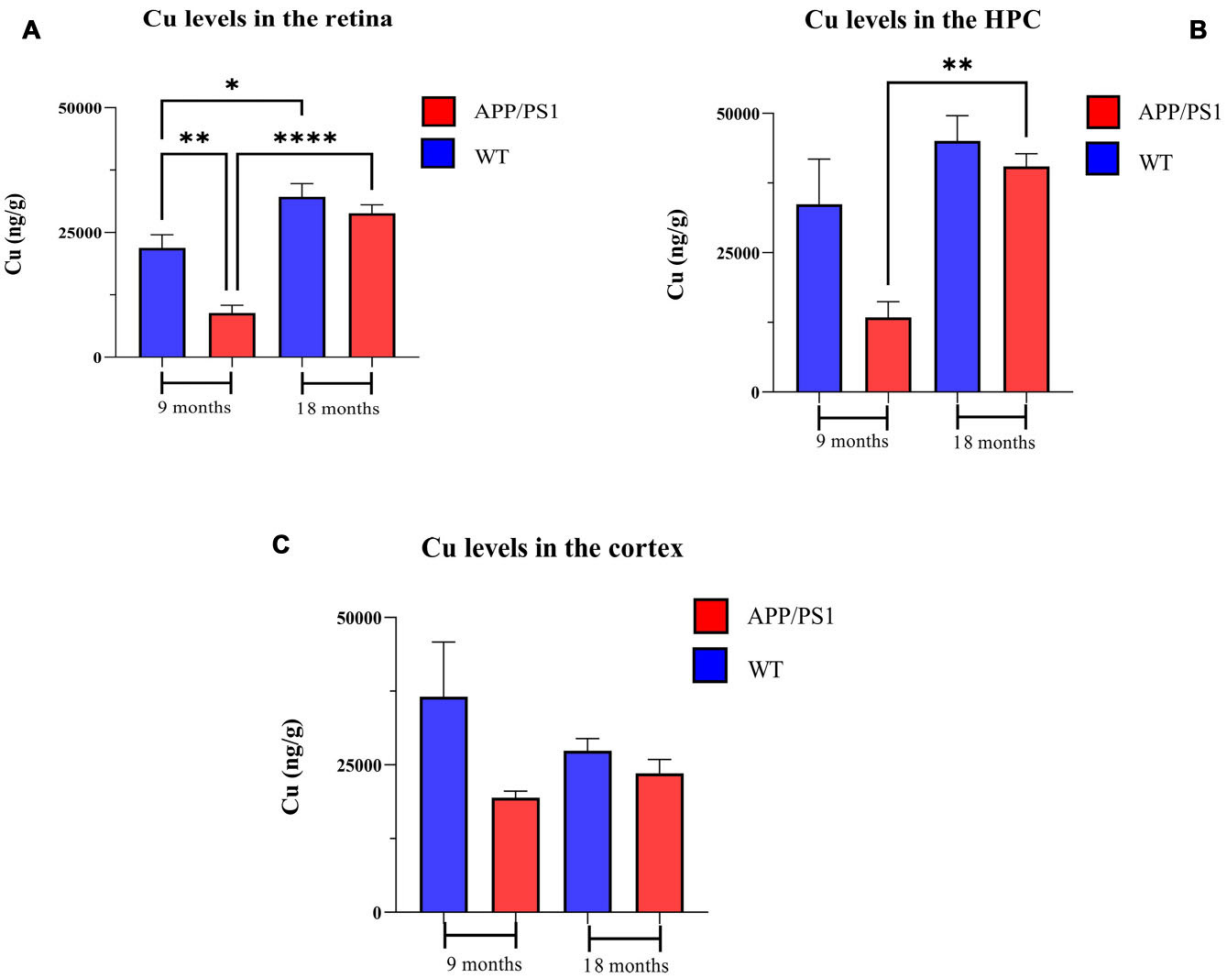
Copper levels in the brain and retina of 9- and 18-month-old APP/PS1 and WT mice. Cu levels in the (A) retina, (B) hippocampus, and (C) cortex. *n* = 10 mice in each group. Error bars represent SEM (*P < .05, **P < .01, and ****P < .0001, Student's *t*-test, unpaired). HPC, hippocampus; CX, cortex.

The Cu concentrations in the retina, hippocampus, and cortex of 9- and 18-month-old mice (APP/PS1 and WT) are summarized in Fig. 1. The overall trend of higher Cu concentrations in the WT mice compared to the APP/PS1 mice was extended from 9 to 18 months of age. In both animal groups, retinal Cu concentrations (ng) were significantly higher at 18 months compared to 9 months of age (8871 ± 4134 *vs* 28 871 ± 5071, *P* < .0001 for APP/PS1 and 21 903 ± 6513 *vs* 32 149 ± 7959, *P* < .05 for WT). Comparison of the retinal Cu concentrations (ng/g) between the APP/PS1 and WT mice revealed a significant difference only at 9 months of age (21 903 ± 6513 *vs* 9585 ± 3348, *P* < .01). In the brain, a 1.33-fold increase in the hippocampus Cu concentration (ng/g) of WT mice was observed from 9 to 18 months of age (33 690 ± 25 638 *vs* 45 049 ± 14 385, *P* = .39). In the cortex, the Cu concentration (ng/g) of WT mice 9 months of age was 1.33-fold higher than 18-month WT (36 565 ± 29 319 *vs* 27 396 ± 6559, *P* = .55).

#### Iron

**Figure 2.**
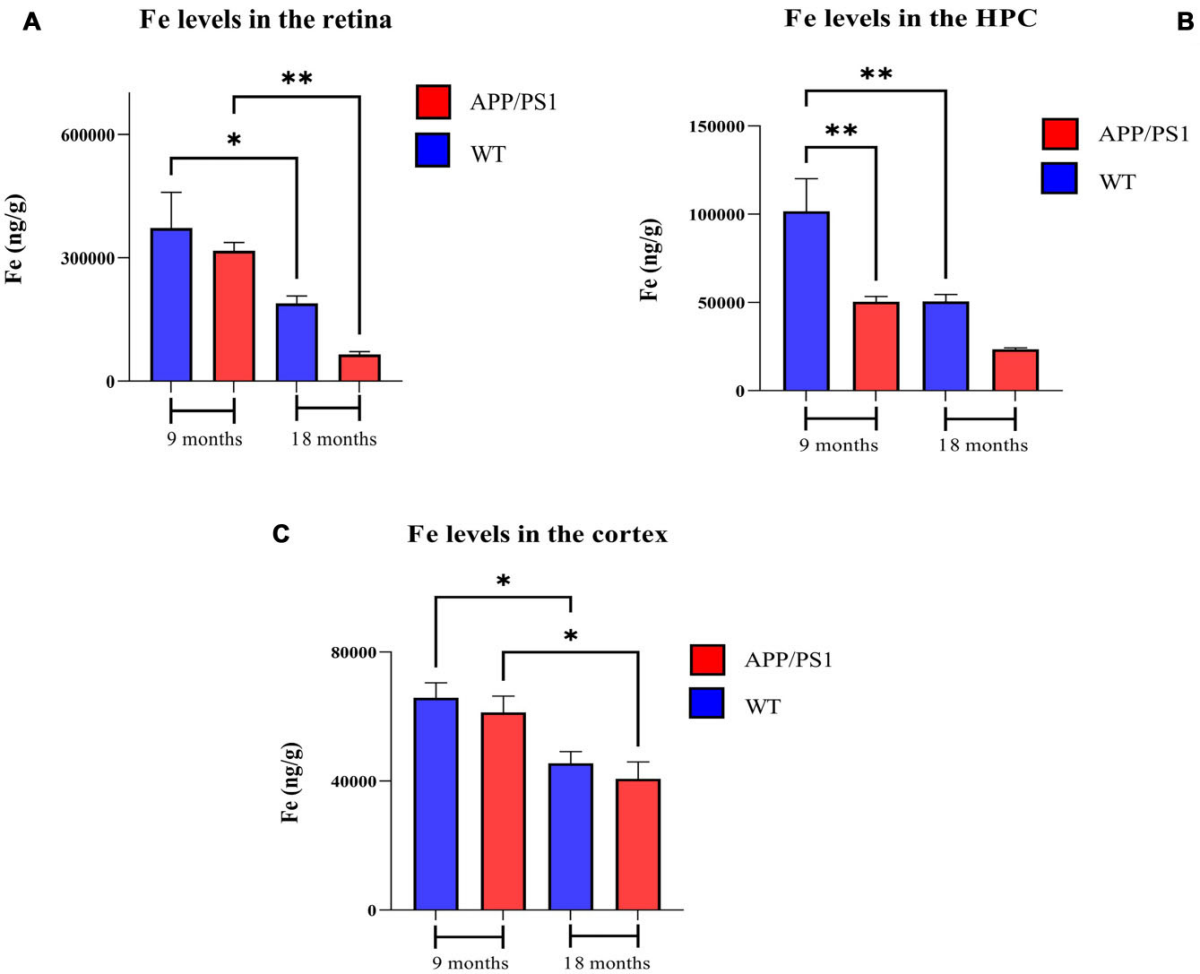
Iron levels in the brain and retina of 9- and 18-month-old APP/PS1 and WT mice. Fe levels in the (*A*) retina, (B) hippocampus, and (*C*) cortex. *n* = 10 mice in each group. Error bars represent SEM (*P < .05 and **P < .01, Student's *t*-test, unpaired). HPC, hippocampus; CX, cortex.

The Fe concentrations in the retina, hippocampus, and cortex of 9- and 18-month-old APP/PS1 and WT mice are shown in Fig. 2. Overall, the Fe levels appeared to decline with aging. In the eye, the retinal Fe levels (ng/g) were higher at 9 months of age compared with 18 months of age (317 173 ± 64 669 *vs* 64 821 ± 22 674, *P* < .01 for APP/PS1 and 372 418 ± 275 448 *vs* 189 435 ± 57 588, *P* < .05 for WT). Comparing hippocampus Fe levels (ng/g) of APP/PS1 with WT mice revealed a significant difference at 9 months of age (101 734 ± 58 281 *vs* 50 405 ± 9580, *P* < .01). The hippocampus Fe levels (ng/g) were significantly higher in the 9-month-old WT mice compared with 18 months old (101 734 ± 58 281 *vs* 50 593 ± 12 567, *P* < .01). In addition, the Fe concentration (ng/g) of AD mice 9 months of age is 1.38-fold higher than 18-month AD (50 405 ± 9580 *vs* 23 496 ± 2635, *P* = .32). Similarly, the Fe levels (ng/g) in the cortex were significantly higher in the 9-month-old WT and APP/PS1 mice compared with 18 months old (65 828 ± 14 723 *vs* 45 528 ± 10 853, *P* < .05 for WT and 61 330 ± 15 166 *vs* 40 695 ± 16 743, *P* < .05 for APP/PS1).

#### Zinc

**Figure 3.**
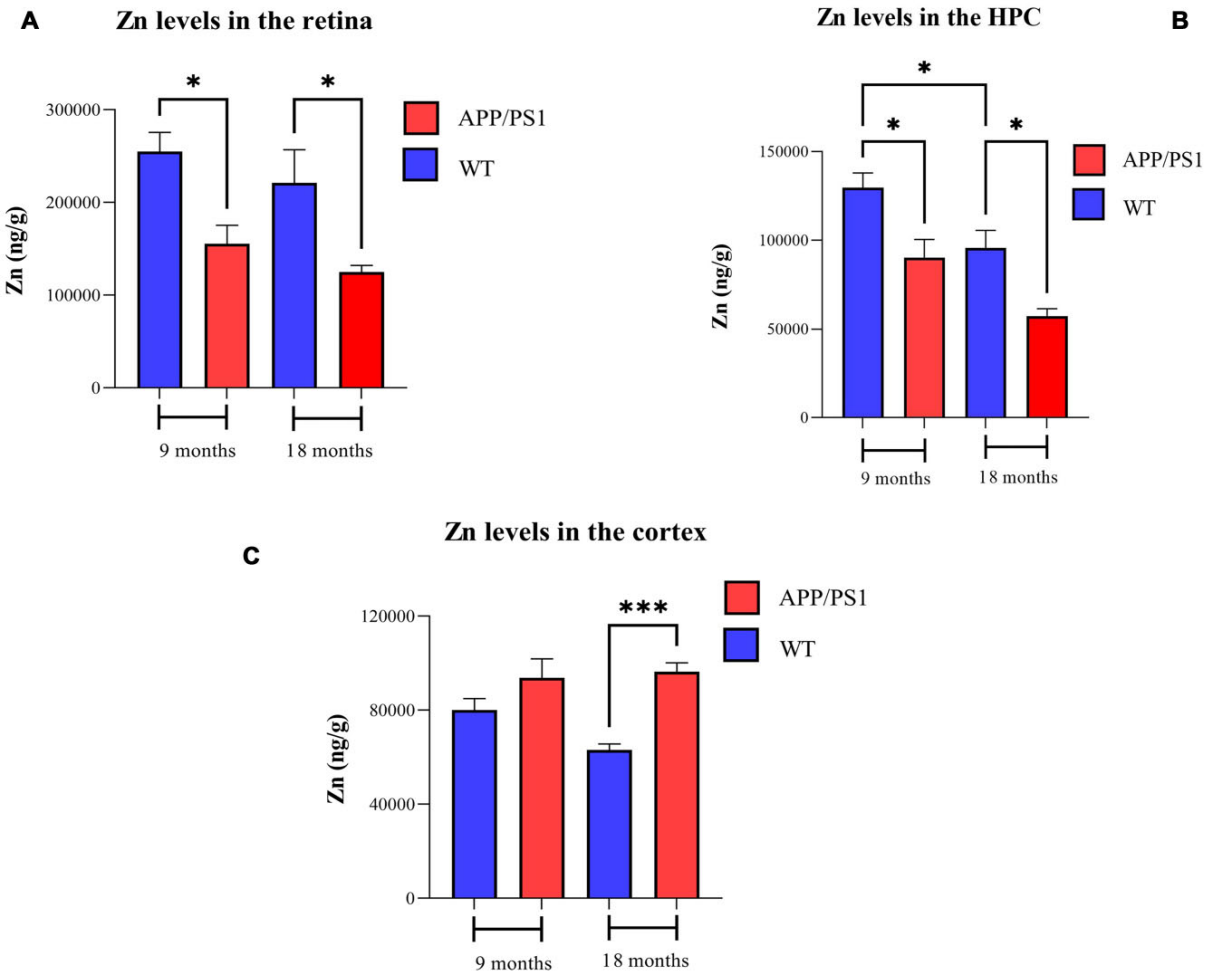
Zinc levels in the brain and retina of 9- and 18-month-old APP/PS1 and WT mice. Zn levels in the (A) retina, (B) hippocampus, and (*C*) cortex. *n* = 10 in each group. Error bars represent SEM (*P < .05 and ***P < .001, Student's t-test, unpaired). HPC, hippocampus; CX, cortex.

Zn concentrations in the retina, hippocampus, and cortex of 9- and 18-month-old APP/PS1 and WT mice are presented in Fig. 3. Similar to Cu and Fe, WT mice possess higher Zn levels than APP/PS1 mice at both 9 to 18 months of age. Comparing eye Zn levels (ng/g) of APP/PS1 with WT mice revealed significant differences at 9 and 18 months of age (254 879 ± 65 664 *vs* 155 452 ± 63 148, *P* < .05 for 9 month and 221 208 ± 112 841 *vs* 124 765 ± 23 418, *P* < .05 for 18 month). In the retina, the Zn concentrations (ng/g) of WT and AD mice 9 months of age are 1.15 and 1.24-fold higher than 18-month WT and AD (254 879 ± 65 664 *vs* 221 208 ± 112 841, *P* = 0.99 for WT and 155 452 ± 63 148 *vs* 124 765 ± 23 418, *P* = 0.99 for AD). The hippocampus Zn levels (ng/g) were significantly higher in the 9-month-old WT mice compared with 18-month-old WT (129 776 ± 26 015 *vs* 95 707 ± 31 509, *P* < .05). In addition, the Zn concentration (ng/g) of AD mice 9 months of age was 1.57-fold higher than 18-month AD (90 260 ± 32 474 *vs* 57 336 ± 13 008, *P* = .06). In the cortex, we observed a decline in Zn levels from 9 to 18 WT, mice display 1.26-fold higher Zn levels than older mice. In the cortex, the Zn concentration (ng/g) in 9-month-old WT mice were 1.26-fold higher than that in the 18-month-old WT mice.

### Evaluation of Fe, Cu, and Zn in the eye and brain (Alzheimer's disease and age-matched healthy human samples)

#### Copper

**Figure 4.**
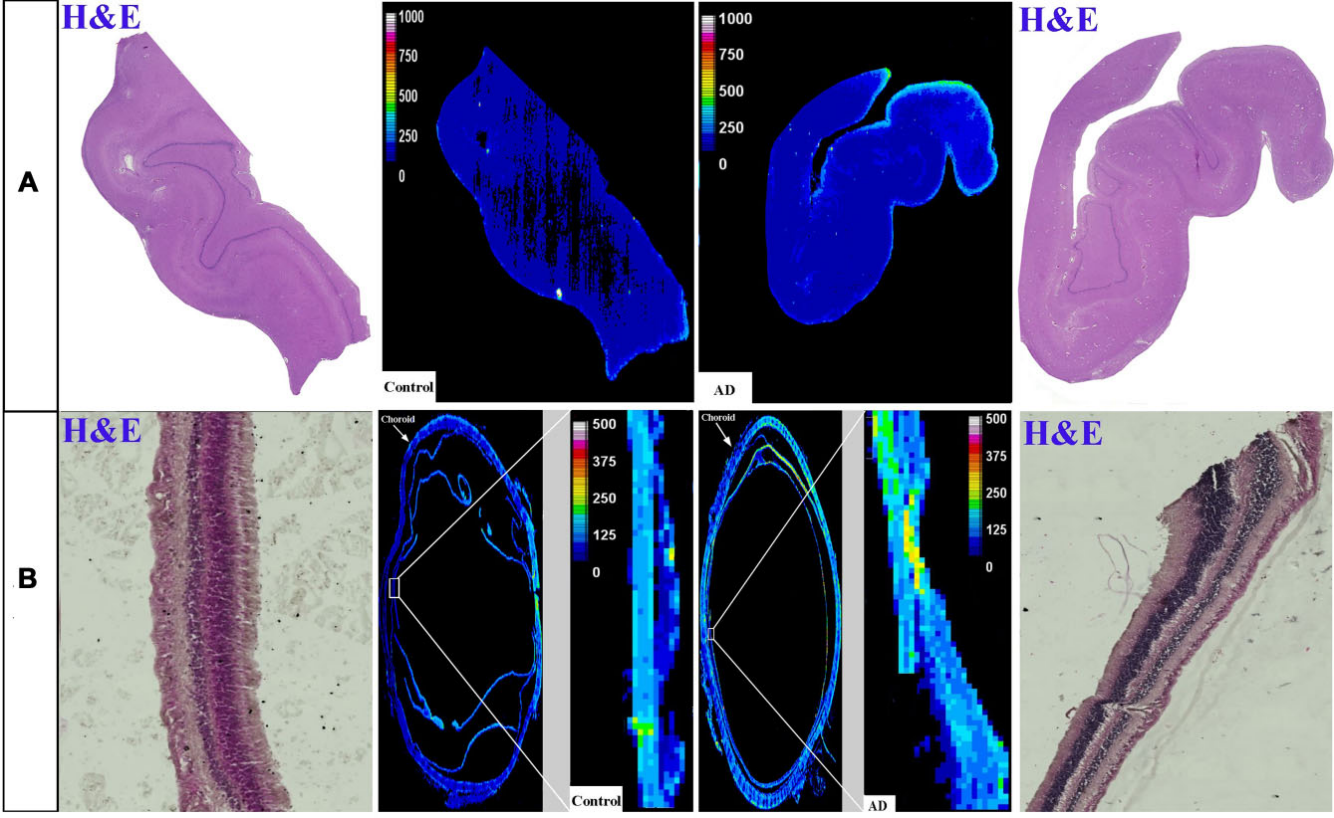
Distribution of Cu in the hippocampus (A) and eye (B). In each panel: *Left and right image*s are representative haematoxylin and eosin (H&E) image demonstrating tissue architecture. *Middle*—sample map of ^63^Cu in a human hippocampus section of healthy control and AD (*upper row*) and a human eye section of healthy control and AD (*lower row*). The scale represents calibrated Cu in ppm.

**Figure 5.**
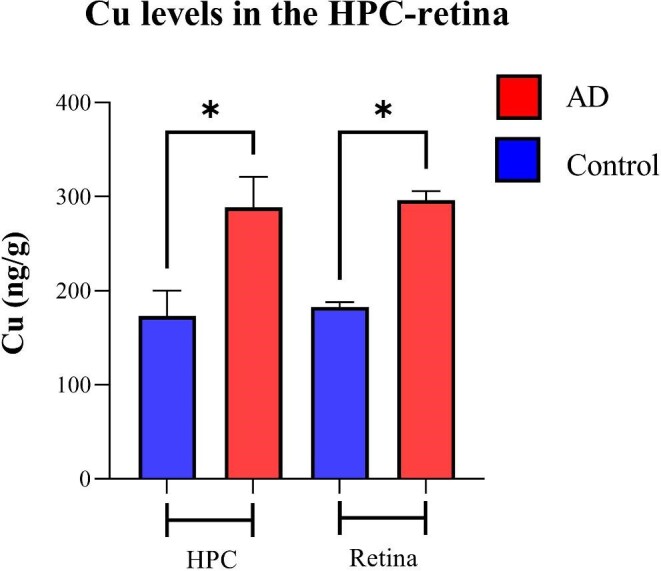
Metal quantification analysis. Cu levels in the retina and HPC of AD and healthy control human samples—nine cases with AD and six healthy controls. Error bars represent SEM (*P < .05, Student's *t*-test, unpaired). HPC, hippocampus.

The distribution of Cu in the human hippocampus and retina of healthy control and AD samples is shown in Fig. 4A and B, respectively. The hippocampus and retina of AD samples display higher levels of Cu compared with the healthy control samples. The hippocampus and retina of the AD samples showed significantly higher Cu levels compared with healthy controls (Fig. 5; 288.3 ± 92.4 *vs* 173 ± 66.8, *P* < .05 for hippocampus and 291.6 ± 29 *vs* 183 ± 9.4, *P* < .05 for retina).

#### Iron

**Figure 6.**
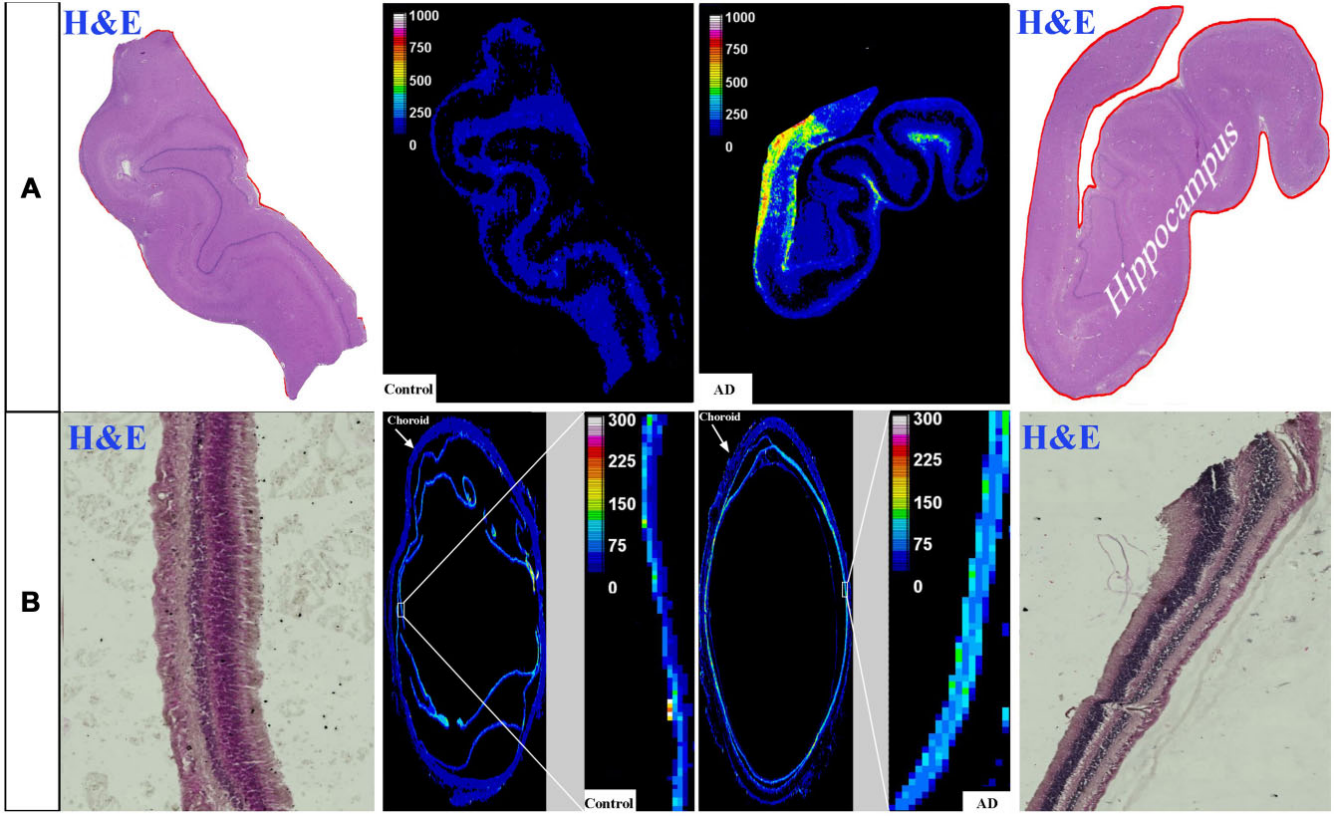
Distribution of Fe in the hippocampus (A) and eye (B). In each panel: *Left and right* images are representative haematoxylin and eosin (H&E) image demonstrating tissue architecture. *Middle*—sample map of ^56^Fe in a human hippocampus section of healthy control and AD (*upper row*) and a human eye section of healthy control and AD (*lower row*). The scale represents calibrated Fe in ppm.

**Figure 7.**
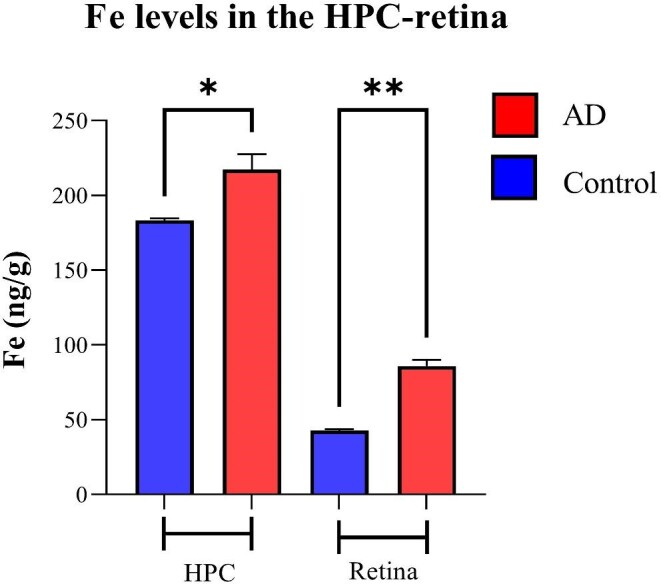
Metal quantification analysis. Fe concentrations in the retina and HPC of AD and healthy control human samples—nine cases with AD and six healthy controls. Error bars represent SEM (*P < .05 and **P < .01, Student's *t*-test, unpaired). HPC, hippocampus.

Figure 6A and B shows the Fe distribution in the human hippocampus and the eye's retina, respectively. Similar to Cu, higher Fe levels (ng/g) were evident in the hippocampus and retina of AD human samples than in the healthy controls (Fig. 6A and B). Figure 7 represents intensity-based analysis results of Fe, which were in accordance with its calibrated quantitative image results; Fe concentration (ng/g) in the hippocampus and retina of AD samples is significantly higher than in healthy counterparts (217.4 ± 26.9 *vs* 183.4 ± 2.9, *P* < .05 for hippocampus and 85.86 ± 12.86 *vs* 42.9 ± 1.3 for retina).

#### Zinc

**Figure 8.**
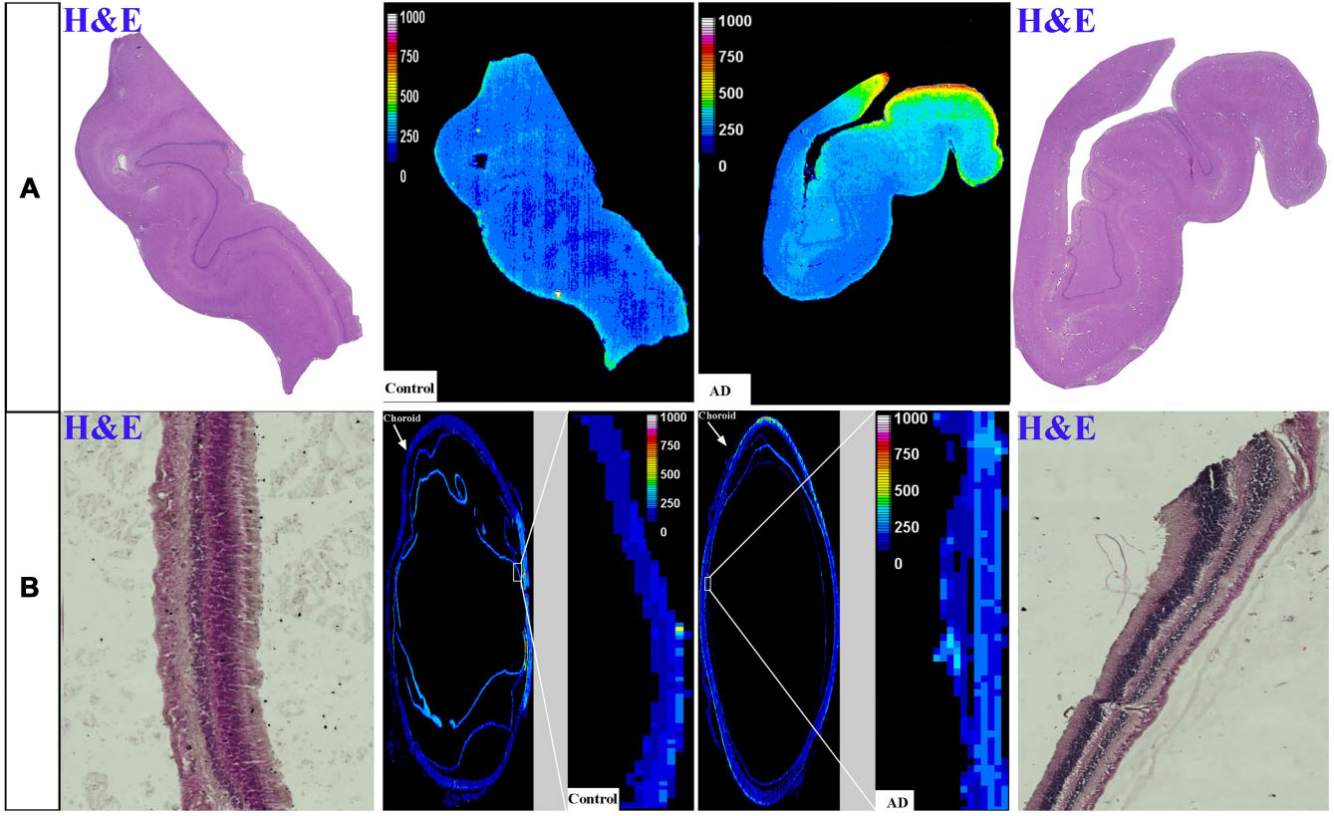
Distribution of Zn in the hippocampus (A) and eye (B). In each panel: *Left and right* images are representative haematoxylin and eosin (H&E) image demonstrating tissue architecture. *Middle*—intensity map of ^66^Zn in a human hippocampus section of healthy control and AD (*upper row*) and a human eye section of healthy control and AD (*lower row*). The scale represents calibrated Zn in ppm.

**Figure 9.**
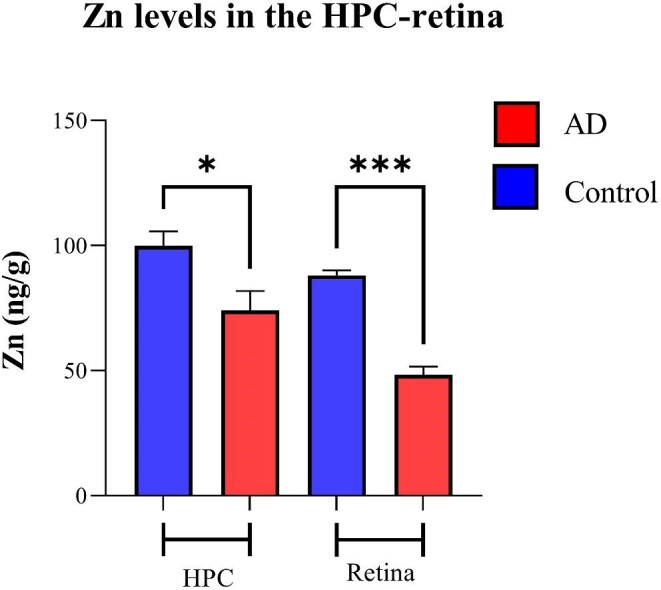
Metal quantification analysis. Zn levels in the retina and HPC of AD and healthy control human samples—nine cases with AD and six healthy controls. Error bars represent SEM (*P < .05 and ***P < .001, Student's *t*-test, unpaired). HPC, hippocampus.

Figure 8A and B shows the anatomical distribution of Zn in the human hippocampus and the retina, respectively. Zn-calibrated quantitative image (Fig. 8A) of the AD hippocampus samples shows higher Zn concentration than the healthy control samples. Similarly, the retina of the AD human sample illustrates higher Zn concentration compared with the healthy control sample (Fig. 8B). The images-based intensity analysis for Zn (Fig. 9) confirms significantly higher concentration of Zn in the hippocampus and retina of AD samples compared with healthy controls (99.7 ± 17.6 *vs* 74 ± 17.4, *P* < .05 for hippocampus and 88 ± 6.1 *vs* 48.3 ± 6.5, *P* < .001 for retina).

### Gender-based evaluation of Fe, Cu, and Zn in the eye and brain (Alzheimer's disease and age-matched healthy human samples)

Gender differences in Zn, Cu, and Fe concentrations in the hippocampus (HPC) and retina of AD patients and post-mortem subjects without AD history were investigated, as shown in Table 2. Comparison of the retinal Cu, Zn, and Fe concentrations between the AD patients and post-mortem subjects without AD history revealed significant differences in both males (M) and females (F). In the brain, only Cu and Fe concentrations showed significant differences between AD patients and without AD history in males.

**Table 2.** Gender differences in Zn, Cu, and Fe concentrations (ng/g) in the hippocampus and retina of post-mortem AD and control subjects.

	Gender	Cu (ng/g)	Fe (ng/g)	Zn (ng/g)
HPC	M	(342.9 ± 122.8 vs 163.0 ± 69.49)*	(200.4 ± 9.232 vs 182.6 ± 2.742)*	(105.1 ± 17.27 vs 78.79 ± 16.03)
	F	(255.6 ± 62.09 vs 223.3 ± 46.53)	(230.1 ± 29.88 vs 186.6 ± 24.29)	(95.50 ± 18.68 vs 55.25 ± 15.25)
Eye	M	(315.8 ± 33.66 vs 180.6 ± 10.09)**	(81.31 ± 16.32 vs 43.37 ± 1.159)*	(84.14 ± 6.656 vs 50.27 ± 6.378)**
	F	(280.4 ± 11.73 vs 190.0 ± 26.53)**	(89.50 ± 9.685 vs 41.50 ± 4.59)*	(91.20 ± 3.756 vs 42.43 ± 7.53)***

*P < .05, **P < .01, ***P < .001, Student's *t-*test, unpaired.

## Discussion

In this study, we utilized LA–ICP–MS and SN–ICP–MS to investigate changes in transition metal levels in the HPC and retina of post-mortem human donors with AD history and post-mortem subjects without AD history, as well as age-related changes in the HPC, cortex, and retina of APP/PS1 and WT mice models aged 9 and 18 months, respectively. Human tissues were obtained from the Netherlands Brain Bank, with only formalin-fixed paraffin-embedded (FFPE) tissue available, while the mice samples were frozen. As a result, we applied two different metal measurement techniques to assess metal concentrations in frozen mice (SN–ICP–MS) and paraffin-embedded human samples (LA–ICP–MS). While the results of these two techniques are not directly comparable due to differences in sample preparation, the observed trends remain valid. Overall, while the specific differences in transition metal levels observed in the animal models were not directly mirrored in the human samples, the trend of differential transition metal concentrations between the retina and brain was consistently observed within each group. The human AD samples exhibited higher levels of Cu, Fe, and Zn compared to healthy controls. The observed order of metal concentrations in our study (Cu > Fe > Zn) deviates from the commonly reported sequence in the literature, where Fe is typically found in higher concentrations than Zn, and Zn is higher than Cu (Fe > Zn > Cu) [37–39]. This discrepancy highlights a significant difference from the conventional understanding and suggests potential influences such as sample handling, tissue type, or analytical methods. Our results, which align with some studies but contrast with the prevalent view, underscore the need for further investigation to reconcile these differences.

In the animal samples, we observed higher transition metal concentrations in WT compared to APP/PS1 mice, except for Zn levels in the cortex, which are higher in APP/PS1 mice than in WT. Cu concentrations demonstrated an age-associated increase, while Fe and Zn levels decreased from 9 to 18 months of age.

Transition metal ions, such as Cu, Zn, and Fe, are key players in modulating Aβ self-assembly in a concentration-dependent manner, where low metal ion concentrations inhibit Aβ fibril formation, while high metal ion concentrations result in amorphous aggregate formation [40]. These metal ions bind monomeric Aβ in the N-terminal part and modulate the Aβ aggregation pathway (vide infra) [40–44]. Furthermore, these metals are also known to modulate tau conformation and enhance its aggregation.

It has been reported that Cu dysregulation instigates and aggravates tau hyperphosphorylation and amyloid plaque formation, eventually leading to synaptic failure, neuronal death, and cognitive decline observed in AD patients [45, 46]. Cu ions may initiate and exacerbate tau hyperphosphorylation by binding to specific fragments of tau (residues 256–273, 287–304, and 306–336) *in vitro* [47–50]. An age-dependent rise in the Cu volume of the HPC has been reported previously [51]. Similarly, our results of age-dependent Cu levels showed an increase in Cu content, in both animal strains, from 9 to 18 months of age; however, Cu levels remained lower in APP/PS1 mice compared with WT mice at each time point. The higher Cu levels could be a result of increasing the expression of Cu transporters or intracellular binding proteins, which ends in increasing Cu uptake or Cu intracellular binding. On the other hand, an increase in intracellular Cu content is caused by the decrease in cellular Cu efflux [51]. The intracellular storage of Cu is regulated by metallothioneins (MTs) [52–54]. Numerous studies indicate that increased MT expression is the cell's protective response to excess Cu, helping to safeguard against the cytotoxic effects of redox-active Cu ions [55, 56]. CTR1 and DMT1 proteins are involved in transporting Cu out of the brain, moving it from the cerebrospinal fluid (CSF) to the bloodstream [57]. Recent studies on sheep have shown a reverse transport of Cu from the blood into the choroidal epithelia [55]. Therefore, the higher Cu levels observed in the aging human brain might be due to dysregulated CTR1 function at the brain barriers [51]. Additionally, age-related changes in DMT1 have been reported, with studies showing a significant increase in DMT1 expression as aging progresses [58, 59].

In contrast to our animal studies, where we observed lower Cu levels in APP/PS1 mice compared to controls (in both the HPC and retina), our human AD samples exhibited higher Cu concentrations in their HPC and retina compared to healthy controls. This finding contradicts previous studies conducted on human samples, which consistently reported significantly lower Cu levels in AD samples [60, 61]. The underlying cause for this discrepancy is likely multifaceted, stemming from the diverse pathophysiological profiles across different population samples. However, it is worth noting that the presence of the epsilon4 allele of the apolipoprotein E gene, known to increase an individual's risk for late-onset AD, has been associated with higher serum Cu concentrations [62, 63]. Consequently, impaired plasma Cu regulation may contribute to elevated brain Cu levels in AD [64–67]. Apolipoprotein E4 (apoE4), the most prevalent genetic risk factor of AD, which is expressed in more than half of AD patients, exhibits a higher affinity for Cu through its four-helix bundle of the N-terminus binding site [68]. The meta-analysis conducted by Squitti and colleagues [69] indicates that elevated levels of ‘free Cu’ in serum lead to an increase in Cu levels in AD patients. While this could potentially explain our findings, we acknowledge the absence of information regarding the genetic disposition of our donor human samples, which precludes definitive verification.

Fe is the most abundant transition metal in the human body [70], which is vital for the metabolic processes of tissues with high oxygen consumption, such as the brain [71]. Fe misregulation within the brain causes oxidative stress and inflammatory responses, leading to cell death and, eventually, neurological diseases such as AD [72]. Fe^2+^ can promote tau phosphorylation by activating cyclin-dependent kinases-5 (CDK5) and glycogen synthase kinase-3β (GSK-3β) [73]. Raising the brain Fe level is known as a feature of normal aging and is further elevated in AD [71]. Increasing Fe concentration has been reported with aging [74, 75]; however, we observed an age-associated decrease in Fe levels; 9-month-old retina and HPC possess higher Fe levels than 18-month-old in both the WT and APP/PS1 mice. This contradictory finding could be due to age-associated overexpression of Aβ42 and lower expression of Fe regulatory proteins in AD, leading to decreased Fe content [76]. The overexpression of the APP C-terminal fragment C100 and the carboxyl-terminal fragment of APP, which contains Aβ, leads to a significant reduction in Fe levels in the mouse brain throughout their lifespan, possibly due to a direct interaction with Aβ [55]. Additionally, Fe homoeostasis becomes imbalanced in the aging brain, with changes in Fe regulatory proteins being more pronounced in AD. Transferrin (TF) secretion into the CSF may be affected during conditions of Fe imbalance [77]. The reduction in TF levels may suggest decreased Fe transport and subsequent utilization within the brain [78]. In addition, a decline in TF levels may lead to reduced Fe transport to neurons, potentially causing metabolic dysfunction [77]. Changes in transferrin receptor protein-1 (TfR1) expression and function, which are affected by aging, can lead to dysfunction in Fe transport within the brain [79, 80].

Unlike the animal results, which demonstrated higher Fe concentration in the HPC and retina of WT than APP/PS1 mice, human AD samples (HPC and retina) possess higher Fe concentration than healthy samples. Similar to our results, higher Fe concentrations in human AD samples than in healthy controls have been reported [60, 81, 82]. The disorder in Fe uptake arises from furin and Fe-regulating elements, and the Fe elimination process may result in higher Fe concentrations in human AD patients [83, 84]. Since furin is responsible for the process that releases soluble hemojuvelin (s-HJV) [85, 86], and Fe overload reduces s-HJV production due to modulation of the FUR (FES upstream region) promoter activity during changes in intracellular Fe concentration [87], furin plays a crucial role in regulating intracellular Fe [87]. Furthermore, it has been reported that also AD brains have a higher level of ferritin than healthy control brains; they possess more Fe content in their ferritin than healthy brains [88]. An Fe fraction is bound to lactoferrin, which localizes to extracellular amyloid and NFT [89]. Therefore, brain regions with abundant NFTs and senile plaques contain high levels of Fe [60].

Zn, the second most abundant d-block metal in the human body, is necessary for brain tubulin growth, phosphorylation, and axonal and synaptic transmission [90]. Zn alterations have been proposed as a risk factor for depression, AD, aging, and other neurodegenerative disorders, with Zn dyshomoeostasis leading to synaptic and memory deficits and the aggregation of β-amyloid protein into neurotoxic amyloid plaques, a key pathological hallmark of AD [91]. Zn can directly bind to tau monomers and stimulate tau protein phosphorylation by activating GSK-3β, ERK1/2, and c-Jun N-terminal kinase. Besides, it induces protein phosphatase 2A inactivation and tau hyperphosphorylation through the Src-dependent pathway, leading to a net increase in phosphorylated tau that may exacerbate AD-like tau pathologies [92, 93]. We observed a significant decrease in Zn volume followed by moving from 9 to 18 months of age in both animal strains, which is consistent with a similar study [94] that mentioned an age-dependent increase in metallothionein-3 (MT3) [95, 96] and a predominant Zn-binding protein in the brain [97] as the reasons for reducing the Zn volume [98]. MT3 has been shown to inhibit abnormal neuronal growth and the formation of NFTs [99]. MT3 shows abnormal expression in AD, as confirmed by immunohistochemistry, northern blotting, and reverse transcription polymerase chain reaction [100]. In mouse brains, MT3 was found in higher concentrations after 12 weeks of age, with a significant increase at 16 months in the HPC [101–103]. In the aging brain, sustained elevated levels of inflammatory cytokines Interleukin-1 (IL-1) and Interleukin-6 (IL-6) trigger ongoing gene expression of MTs, which restricts Zn release in response to intracellular Zn signals [104]. Overexpression of MT in sheep pulmonary artery endothelial cells results in the inhibition of NO-mediated Zn release, which can be restored by growing the cells in media with high Zn concentrations [105]. Age-related elevations in MT3 lead to the suppression of various Zn-dependent biological processes, including metabolism, gene expression, and signal transduction [106]. Studies have shown that, during prolonged stress, rats have higher levels of Zn-MTs in their brains [107], which results in these proteins taking Zn away from brain cells or brain Zn transport proteins (ZnT1–T4) [108]. This process likely explains why the amount of Zn is lower in the mossy fibres of the HPC in older rats compared to younger adult rats [104]. In addition, in old mice, high IL-6 levels and MT mRNA expression have been related to low Zn ion bioavailability [109]. Abnormally high levels of brain Zn-MTs during aging could be harmful due to their ability to potentially deplete Zn from within neurons. Thus, Zn supplementation during aging can increase Zn ion bioavailability by promoting faster degradation of Zn-MTs, preventing the continuous depletion of intracellular Zn by these proteins [110]. The accelerated aging seen in individuals with Down's syndrome is characterized by reduced Zn levels, impaired brain function, and increased expression of Zn-MTs [111]. Together, these findings further prove the role of MT in regulating Zn levels.

Contradictory to our animal findings, in our human samples, we found higher Zn concentration in the AD donor samples (HPC and retina) than in healthy controls. Similarly, previous studies on human samples have reported higher Zn levels in AD than in healthy controls [60, 81, 112, 113]. High levels of Zn-enriched neuron (ZEN) terminals [112, 114] and low concentration of ZnT3 transporter in AD can explain the high Zn levels we have observed [115]. In the Central Nervous System (CNS), substantial amounts of Zn ions and ZnT3 proteins are found in the ZEN terminals [114]. The high level of Cu–Zn superoxide dismutase (SOD1) was observed in regions heavily affected by AD, which could explain the elevated Zn concentration in the AD brain [60]. Religa *et al*. [113] reported that Zn concentrations in the AD brain were twice as high compared to healthy controls, which was linked to increased Aβ accumulation and greater overall AD severity.

The discrepancy between our findings in animal models and human samples can be attributed to several factors: (i) Although mice and humans share 92% of their DNA, mice serve as ideal models for only certain human diseases [116]. To accurately model complex diseases like AD, which involve multiple genomic variants, it is necessary to replicate the exact human mutated genes in the mouse genome. Moreover, APP/PS1 mice used in our study are primarily used to model familial AD, while the human samples in this study were confirmed to have the sporadic form. (ii) Despite the human and mouse brain comprising similar cell types, including neurons, significant differences exist in the expression of individual genes within the same cells. A notable discrepancy is observed in neurons, where genes responsible for producing serotonin receptors are active in mice but inactive in humans. Serotonin levels have been identified as potentially beneficial for AD prevention and treatment [117]. It has been reported that heavy metals such as Cu, Zn, and Fe result in neuroinflammation, oxidative stress, hormone fluctuation, and neurotransmitters perturbation such as dopamine and serotonin [118–120]. The levels of Cu, Zn, and Fe are higher in WT mice than in APP/PS1 mice, which results in higher levels of serotonin. (iii) Unlike humans, mice have the ability to synthesize their own vitamin C. There is substantial evidence indicating that vitamin C plays a protective role against age-related cognitive decline and AD, and there is a significant decrease in the plasma vitamin C levels of AD patients than healthy controls [121–123]. Vitamin C is known to enhance the absorption of Fe and Zn [124, 125]. However, the effects of ascorbic acid on Cu absorption are not conclusive [126]. Our WT mice expressed higher Fe and Zn concentration than AD mice, which could be due to the high level of vitamin C. Collectively, it is plausible to assume that our APP/PS1 and WT mice models do not accurately reflect the same distribution and variations of metals found in human brain tissues.

## Limitations

Despite the current study being the first to objectively quantify transition metal levels in both human and animal model eye and brain samples, our study has several limitations. Firstly, the potential for contamination during the extensive sample preparation process could significantly influence metal concentration readings. Secondly, slicing samples at an angle to the blade might result in images that do not fully capture the true representation, potentially skewing the quantified metal levels away from the actual physiological content [127]. Thirdly, the use of transgenic animal models, which are genetically engineered, may not perfectly encapsulate the multifaceted origins of the most prevalent form of AD/sporadic AD [128]. The progression of AD in these models also unfolds within a markedly different timeframe compared to human patients [129]. Finally, for human samples, the formalin fixation process poses a risk of significant leaching of trace elements from tissues, an aspect that necessitates careful consideration [130]. A loss of metals in chemical fixation process could be explained by the fixation process and subsequent washing, which allow the escape of ‘labile’ metals. It is reasoned that immersing the tissue sections in aqueous medium results in the exchange or loss of extra- and intracellular fluid, along with the corresponding matrix of water-soluble molecules, ions, and trace metals, to the surrounding medium [131, 132]. The control group in our study experienced the same sample preparation protocols and potential metal loss as the AD samples. As a result, any biases or losses due to sample preparation would be consistent across all groups, allowing for meaningful comparisons between them. Although absolute metal concentrations might be affected, the trends we observe between the groups remain valid.

## Conclusion

This study marks a pioneering effort to explore age-related changes in the distribution and concentration of transition metals (Cu, Fe, and Zn) in the brain and retina across both human subjects and the APP/PS1 mouse model of AD. In a departure from the trends observed in animal studies, where WT models displayed higher levels of these metals compared to their AD counterparts, human samples obtained from AD donors showed elevated levels of transition metals compared with healthy controls. Our findings support the notion that pathological alterations associated with AD in the brain are mirrored in the eye. However, further research is necessary to determine whether these observations are specific to certain forms of AD (such as familial or sporadic) or are a universal phenomenon. Importantly, our results underscore the potential of assessing transition metal levels in the retina as a means to identify early-onset AD in the brain, opening new avenues for diagnosis and understanding of this complex condition.

## Data Availability

The data underlying this article will be shared on reasonable request to the corresponding author.
